# Children and Adolescents with Neurodevelopmental Disabilities: Ecological Assessment Tools and Cognitive Analysis

**DOI:** 10.3390/children13010073

**Published:** 2026-01-02

**Authors:** Yael Fogel, Naomi Josman

**Affiliations:** 1 Department of Occupational Therapy, Ariel University, Ariel 4070000, Israel; 2 Department of Occupational Therapy, Faculty of Social Welfare and Health Sciences, University of Haifa, Haifa 3498838, Israel; njosman@univ.haifa.ac.il

The Special Issue, “Children and Adolescents with Neurodevelopmental Disabilities: Ecological Assessment Tools and Cognitive Analysis,” brings together a diverse collection of articles that highlight the rapidly evolving landscape of assessment, participation, and cognitive analysis in children and adolescents with neurodevelopmental disabilities (NDDs). As guest editors, we are grateful to the authors, the reviewers, and the *Children* journal for contributing to the success of this Special Issue, which now includes a rich set of 11 high-quality papers.

Functional cognition has emerged as one of the most critical domains in understanding children’s and adolescents’ participation in meaningful daily activities [1]. *Functional cognition* reflects the dynamic interplay of cognitive abilities, performance skills, environmental demands, and task requirements—elements that traditional, decontextualized cognitive tests cannot fully capture [2,3]. Over the past decade, the field has seen a conceptual shift: from focusing primarily on isolated cognitive components (bottom-up) toward emphasizing real-world functional performance (top-down) [4]. This shift aligns with broader occupational therapy and neuropsychological frameworks that emphasize ecological validity, contextual performance, and strategy use as essential indicators of meaningful cognitive functioning [5].

In our conceptual review on digitizing functional cognitive assessments, we found that although technology offers major opportunities, such as precise measurement, accessibility, and scalability, most performance-based assessment tools with the strongest ecological validity remain unavailable in digital formats.

The review demonstrates a persistent gap: Tools that best capture real-life functioning (e.g., complex cooking tasks, calendar planning, and naturalistic assessments) are precisely those most challenging to translate into digital platforms without compromising the richness of observation or strategy identification. At the same time, emerging technologies, such as virtual reality (VR), mobile platforms, and algorithm-driven adaptive assessments, show promise in bridging this gap by offering controlled yet naturalistic environments that can simulate real-life complexity. This technological evolution underscores the need for advanced digital tools that maintain representativeness, contextual fidelity, and the ability to capture strategy use—all hallmarks of ecologically valid functional cognitive assessment [6].

Collectively, the contributions to this Special Issue highlight a movement toward integrated assessment approaches that view cognitive abilities not as isolated mechanisms but as embedded within daily participation, emotional regulation, social interaction, and task demands. The articles span a wide range of populations (attention-deficit/hyperactivity disorder [ADHD], autism spectrum disorder, traumatic brain injury, genetic syndromes, learning disabilities, and typically developing youth). They present tools that assess executive functions, memory, attention, problem-solving, and self-regulation using real-world or simulated tasks. Many contributions also integrate intervention perspectives, reflecting the growing understanding that assessment and treatment are interdependent—particularly within dynamic, context-sensitive frameworks.

A clear conceptual thread emerges across the Special Issue: the transition from bottom-up assessments, which assess cognitive components in isolation, toward top-down models, which evaluate performance within meaningful occupations. This transition is evident in the tools that examine participation, naturalistic behavior, everyday task complexity, and functional problem-solving. The studies collectively emphasize that understanding performance in real contexts is indispensable for accurately identifying strengths, challenges, learning potential, and intervention needs among children and adolescents with NDDs [7].

## 1. Future-Oriented Research Directions Across the Contributions

The collective body of work in this Special Issue highlights important opportunities for advancing the field of functional cognition. First, there is a clear need to continue developing ecologically valid digital assessment tools that preserve the richness of real-world observation while leveraging technological precision and scalability. Future studies should explore hybrid models that integrate naturalistic tasks with digital data capture, including VR-based simulations, sensor-supported monitoring, and adaptive mobile assessments. Second, there is a growing call for longitudinal research examining how functional cognition evolves across developmental stages and contexts, particularly in relation to participation, autonomy, and learning potential. Third, the contributions underscore the importance of cross-cultural validation and normative data for performance-based assessments, ensuring that assessment tools are sensitive to diverse environments and populations. Finally, future research should deepen the connection between assessment and treatment by investigating how performance-based measures can directly inform individualized strategy training, classroom accommodations, and family-centered support. Together, these directions point toward a dynamic, interdisciplinary future that seamlessly integrates ecological assessment, technological innovation, and participation-focused intervention.

## 2. An Overview of the Published Articles

### 2.1. Diagnosis Identity Perception in Adolescents with ADHD and Its Relationship to Executive Functions, Self-Management, and Quality of Life (C1)

This article explores how adolescents perceive their ADHD diagnosis and how this identity relates to executive functioning, self-management, and quality of life. The findings reveal that negative diagnosis identity predicts poorer executive function performance and reduced participation, highlighting the interplay between emotional experience, metacognition, and real-life functioning. This study contributes a unique psychosocial dimension to functional cognition, showing that identity and daily participation are intertwined. The study bridges assessment and intervention by pointing to the need for holistic, context-sensitive support.

### 2.2. Occupation-Based Tele-Intervention for Children with Neurodevelopmental Disorders: A Pilot Study (C2)

This pilot study adapts the cognitive orientation to daily occupational performance model for telehealth delivery, demonstrating meaningful functional gains in daily occupations. By focusing on self-selected goals and metacognitive strategies, the intervention aligns with top-down, participation-based assessment and treatment. Parents and therapists reported that working in the child’s natural home environment enhanced generalization—emphasizing the ecological strengths of tele-intervention. The study illustrates how assessment and intervention are interdependent processes shaped by real-life contexts.

### 2.3. Assessment of Fine Motor Abilities Among Children with Spinal Conditions (C3)

The authors evaluate fine-motor performance in children with spinal disorders using functional, activity-focused tools that capture real-life task execution (e.g., handling objects, manipulating materials). The results highlight significant gaps between impairment-level findings and actual performance in daily occupations, demonstrating the limits of bottom-up motor assessments. This article reinforces the need for ecologically valid, occupation-centered tools to understand how motor deficits influence participation and independence. Its focus on naturalistic performance connects directly to the Special Issue’s emphasis on functional cognition.

### 2.4. Effects of Medical Cannabis Treatment for Autistic Children on Family Accommodation: An Open-Label Mixed-Methods Study (C4)

This mixed-methods study shows that cannabidiol-rich cannabis treatment reduced family accommodation behaviors and parental distress while improving family routines and participation. Although medical treatment is not a cognitive intervention per se, the findings demonstrate how reductions in behavioral rigidity can enhance functional participation within daily occupations. The study emphasizes the family context as a critical ecological layer influencing a child’s functional cognition. It reinforces the relevance of participation-based outcomes as essential indicators of meaningful change.

### 2.5. Independence in Activities of Daily Living Among Autistic Toddlers: A Pilot Study Using Ecological Momentary Assessment (C5)

Using ecological momentary assessment, this study captures toddlers’ daily living skills across real-time contexts. Results show that autistic toddlers demonstrate greater independence at home than indicated by one-time clinic assessments, highlighting the limitations of decontextualized evaluations. Ecological momentary assessment provides a rich, ecologically grounded picture of functional cognition, capturing variability, environment–task interactions, and natural performance. This article strongly supports the Special Issue’s emphasis on real-world assessment and the top-down perspective.

### 2.6. ADHD Reporting in Developmental Age: The Role of the Informants (C6)

This article examines patterns of ADHD-symptom reporting across developmental stages using multi-informant perspectives. The study highlights the importance of contextualized behavioral observations, showing discrepancies among teachers, parents, and clinicians that reflect setting-dependent executive function performance. The findings align with functional cognition principles, illustrating that ADHD-related behaviors cannot be fully understood through isolated, component-based assessments. The paper underscores the need for ecologically grounded evaluations that consider context, participation demands, and daily routines.

### 2.7. Developmental Patterns in Autism and Other Neurodevelopmental Disorders in Preschool Children (C7)

This study analyzes developmental and adaptive profiles across autism spectrum disorder, language disorder, and mixed developmental disorder using multiple standardized tools and caregiver reports. Results confirm highly heterogeneous trajectories, demonstrating that diagnostic labels poorly generalize to functional performance in daily life. The authors show that caregiver-based ecological assessments add essential insight beyond clinic-based testing—especially regarding communication, motor skills, and adaptive functioning. This aligns strongly with the Special Issue’s goal of understanding cognition within natural environments and across diverse NDD populations.

### 2.8. A Virtual Reality Platform for Evaluating Deficits in Executive Functions in Deaf and Hard of Hearing Children: Relation to Daily Function and to Quality of Life (C8)

This study introduces a VR-based assessment (supermarket) designed to simulate everyday executive-function challenges in a controlled yet ecologically valid environment. Findings show that VR tasks successfully capture real-world behaviors, such as planning, flexibility, and inhibition, beyond what traditional paper-and-pencil tests can measure. The virtual supermarket reflects the field’s movement toward digital, performance-based assessments that preserve contextual demands. By replicating complex tasks in a safe virtual environment, the study demonstrates how technology can bridge the gap between top-down functional evaluation and quantifiable cognitive metrics.

### 2.9. Can Functional Cognitive Assessments for Children/Adolescents Be Transformed into Digital Platforms? A Conceptual Review (C9)

This conceptual review examines the feasibility and implications of digitizing performance-based functional cognitive assessments for children and adolescents. Reviewing 13 assessment tools, the authors show that most high-ecologically valid assessments remain nondigital, whereas existing digital tools often sacrifice contextual realism. The article highlights a major gap between clinical need and technological development and emphasizes that digital platforms must preserve real-world context, strategy observation, and process-oriented evaluation. It proposes a guiding conceptual framework to support future development of ecologically valid digital assessments. The review concludes with recommendations for leveraging VR, mobile devices, and advanced analytics to enhance the precision, accessibility, and scalability of functional cognitive evaluation.

### 2.10. Sleep Disorders in Children with Rett Syndrome (C10)

This review synthesizes evidence on sleep disturbances among children with Rett syndrome, a population in which approximately 80% experience significant sleep difficulties. The article details common issues, such as insomnia, abnormal nocturnal breathing, parasomnias, bruxism, and seizures, and examines genotype–phenotype associations. Objective findings from polysomnography reveal reduced sleep efficiency, elevated arousals, disrupted REM/NREM patterns, and both central and obstructive sleep apnea. Recommendations for management emphasize behavioral sleep interventions, careful pharmacological consideration due to autonomic and cardiac vulnerabilities, and the importance of individualized multimodal care. The authors call for more rigorous research and registry-based studies to advance evidence-based treatment strategies.

### 2.11. XR Technologies in Inclusive Education for Neurodivergent Children: A Systematic Review 2020–2024 (C11)

This systematic review synthesizes evidence on extended reality tools used to support neurodivergent students in inclusive education. The findings emphasize improvements in engagement, social communication, and cognitive participation within simulated learning environments. Extended reality emerges as a promising pathway for creating controlled yet realistic contexts that mirror daily classroom demands. The review highlights the potential of extended reality to bridge digital assessment with real-world participation, supporting the Special Issue’s theme of emerging technologies in functional cognition.

## 3. Authentic, Integrated, and Person-Centered Approaches

Collectively, the articles in this Special Issue advance the field toward more authentic, integrated, and person-centered approaches. They reinforce the importance of aligning assessments with real-world contexts and of acknowledging the rich diversity of children’s experiences, abilities, and environments. It is our hope that this Special Issue will inspire continued innovation and collaboration across clinical, educational, community, and research settings.

We extend our sincere appreciation to all authors whose work contributed to this Special Issue and to the *Children* editorial team for their support and professionalism. We look forward to seeing how the ideas presented here will influence future research, shape practice, and expand our collective understanding of children and adolescents with neurodevelopmental disabilities.
